# Inequity in palliative care and bereavement support: an umbrella review of the evidence

**DOI:** 10.3389/fpubh.2026.1943244

**Published:** 2026-10-19

**Authors:** Annie Pettifer, Muzeyyen Seckin, Nikolaos Efstathiou, Ping Guo, Rumandeep Tiwana, Cara Bailey

**Affiliations:** 1 Department of Nursing and Midwifery, School of Health Sciences, College of Medicine and Health, University of Birmingham, Birmingham, United Kingdom; 2 St Giles Hospice, Lichfield, Staffordshire, United Kingdom

**Keywords:** bereavement, death and dying, disadvantage, inequality, inequity, palliative care, umbrella review

## Abstract

**Introduction:**

Inequitable, unfair or unjust service provision pervades palliative and bereavement care globally within and across countries. There is growing research into the needs of population groups known to experience inequity. This paper presents an evidence synthesis of systematic reviews examining inequities in palliative care and bereavement support for adults, to identify the key facilitators, barriers, and structural determinants of inequity across population groups, and to develop evidence-informed recommendations for policy, education, research, and practice.

**Methods:**

Joanna Briggs Institute Umbrella review methodology was used. Searches were conducted across six electronic databases (CINAHL, Medline, PsycINFO, Scopus, Embase, and Cochrane Collaborative Database of Systematic Reviews) from January 2012 to November 2023 and updated the search in May 2025. Joanna Briggs Institute critical appraisal tools were used for quality assessment.

**Results:**

Forty-eight reviews were included. Only 1 review focused exclusively on bereavement, a further 2 studies included it within a broader scope. Barriers and facilitators to equity were identified at individual, population-specific, and system-wide levels. Common structural determinants across all groups included low socioeconomic status, limited health literacy, geographical and structural impediments, cultural and language dissonance, and individual vulnerability due to interconnected personal characteristics. Key recommendations to improve equity included enhancing cultural competence, inclusive communication, intersectional awareness, person-centred care, shared decision-making, and community engagement.

**Discussion:**

Inequity in palliative care and bereavement support reflect collective and shared experiences. The structural determinants of inequity that transcend population groups may be useful in identifying, assessing and measuring inequity in palliative care and bereavement support and aid the commissioning, delivery and evaluation of inclusive services.

## Introduction

Inequitable, unfair or unjust service provision pervades palliative care and bereavement support globally. Over 73.5 million people, mostly in low- and middle-income countries, are estimated to have needed palliative care in 2021, defined as care that provides relief of suffering from life threatening illness and measured using serious health-related suffering, and trends indicate the number is growing (1). Global service provision is not adequate to meet this need (1, 2). Periodic assessment of the palliative care provision worldwide consistently shows considerable inequity of provision particularly related to country income levels. Global ranking undertaken in 2017 showed only 14% of the global population had access to palliative care with more than 50% of the population having severely or entirely limited access despite it being key to achieving Universal Health Coverage (3, 4). Palliative care includes care of those at the end of life and their families.^,5^ Bereavement support for families anticipating or following death of a loved one is an espoused component of palliative care, yet service provision lags that of care of the person (5). To the best of our knowledge, there are no global rankings of bereavement provision.

Inequality of palliative care is evident within countries as well as across them. In a recent global mapping, only 17% of the 201 countries ranked within the WHO framework of palliative care systems were classified as ‘established’, in which palliative care services and policy were pervasive but there were gaps in provision (4). A further 14% of countries were ranked to have ‘advanced’ palliative care with high level of access to services, integration, and sustained policy implementation (4). However, even within ‘advanced’ countries, inequity pervades. For example, while the United Kingdom (UK) ranks as ‘advanced’, groups of people are known to be persistently disadvantaged and receive inequitable palliative care such as people experiencing homelessness; imprisoned people, and lesbian, gay, bisexual and transgender, or queer (LGBTQ) people, those living in remote and rural areas; those who are old, living with frailty and dementia; people living in poverty, deprivation, and with lower socioeconomic status; people living with learning disabilities and people living with non-malignant conditions (6, 7). Successive government policy and legislation has attempted to decrease inequity within the UK but it remains pervasive (8–10). Contributing factors include lack of data about the extent of inequity in service provision, lack of engagement with communities experiencing inequity and lack of understanding about the effects of intersectional disadvantage (7). A synthesis of the current evidence is needed to address this gap.

## Review aim and objectives

This paper presents the findings of a systematic umbrella review which aimed to synthesise evidence from systematic reviews examining inequities in palliative care, and bereavement support for adults. This review is part of a programme of work, undertaken in collaboration with the charity Sue Ryder, to inform understanding of inequity within national adult palliative care and bereavement services, policy and commissioning, which has been published as a report (10, 11). This paper adds international relevance and robust scientific perspectives by providing the conceptual and methodological details and discussion (10). The paper focuses on the following objectives:

1 To explore the extent to which research into inequity in palliative care and bereavement support has directly engaged with the affected population groups.
2 To identify barriers and facilitators contributing to inequity across these population groups.
3 To identify the structural determinants of inequality which span the population groups known to experience inequity
4 To collate existing recommendations for reducing inequity in palliative care and bereavement support through future research, policy, and practice across population groups.

## Methods

### Design

The methodology followed the Joanna Briggs Institute’s guidelines for conducting umbrella reviews (12) and is reported in line with the Preferred Reporting Items for Overviews of Reviews (PRIOR) Statement (13). Umbrella reviews involve the systematic identification, critique, and synthesis of multiple systematic reviews related to a topic or question (14). Although they are limited by the breadth and quality of the existing systematic reviews, they provide a high standard of evidence and offer a broad perspective for addressing issues in healthcare (15, 16). An umbrella review methodology was chosen because there are extensive relevant systematic reviews in the field and their scope is broad because inequity has been studied within and across several population groups. The umbrella review methodology enabled drawing on this existing evidence and systematic interrogation of it with specific objectives that address current challenges.

A protocol for this review was developed and peer reviewed by researchers experienced in systematic reviews. Ethical approval was not sought as data were secondary.

### Search strategy and information sources

A search strategy was developed to identify relevant reviews that systematically describe inequities in palliative care and bereavement support. The search was conducted in six databases: Cumulative index of nursing and allied health literature (CINAHL), MEDLINE, PsycINFO, Embase, Scopus, and Cochrane Collaborative Database of Systematic Reviews. Search terms of key words mapped to subject heading or Medical Subject Headings (MeSH) terms as appropriate to each database, were clustered into two groups combined with the Boolean operation ‘AND’. The two groups were *inequity* using the terms inequit* OR unequit* OR unequal* OR inequal* OR disadvant*, and *Palliative, end of life and bereavement care* using the terms: ‘end of life’ OR end-of-life OR Palliative* OR Terminal OR Hopsic* OR Bereave*. A summary of the Medline search syntax is provided in Supplementary File 1. The primary search was conducted in November 2023, and all database searches were updated in May 2025. The reference lists of selected reviews were also screened.

### Eligibility criteria

The titles and abstracts of the retrieved reviews within the initial search were screened against the agreed eligibility criteria (Table 1) by a single reviewer (AP). The updated search results were screened by MS and checked by AP.

**Table 1:** Eligibility criteria.

Inclusion criteria	Exclusion criteria
Any systematic reviews of any kind, as defined by systematic review authors.	Systematic reviews without a reported methodology No full text located online
Published between 2012 and 2025	Prior to 2012
Adults patients (18+) with a diagnosis of any terminal diseases receiving end-of-life and palliative care and bereavement support. Researched inequity experienced by patients and/or families	Exclusively on care of children (under 18 years of age)
Reviewed primary research on inequity in end-of-life and palliative care and bereavement support singularly or in combination	Focused on death without a palliative phase (such as sudden death) or focused on supportive care only
Was undertaken in any country	
Published in English	Written and published in a language other than English

### Selection and data extraction process

The identified reviews were imported into Covidence (17), an electronic platform that facilitates the management of reviews. Duplicates were identified and removed. Titles and abstracts were screened independently by two reviewers against the inclusion and exclusion criteria. The full texts of reviews whose titles and abstracts met the eligibility criteria were appraised independently by two reviewers with a third reviewer involved in reaching consensus where needed. Following agreement on the inclusion of papers, a data extraction form was developed by AP and CB. It was guided by Joanna Briggs Institute Data Extraction form for Systematic reviews and Research Synthesis (12) and the specific objectives of the umbrella review. The form was piloted by each reviewer and revised accordingly. Data were extracted from each review by allocation to two members of the review team. Any disagreements or conflicts were resolved by discussion. The PRISMA flow diagram (Figure 1) illustrates the process of selecting systematic reviews for inclusion. The data extraction table included the following domains: authors; country; review aim; review design; population (s) facing inequity included in the review; number of studies included in the review; review publication date; quality appraisal checklist used; engagement of people experiencing inequity in the review; factors that increased the risk of inequity; barriers and facilitators affecting access to palliative care and bereavement support and recommendations made in the review to promote equity.

**Figure 1:**
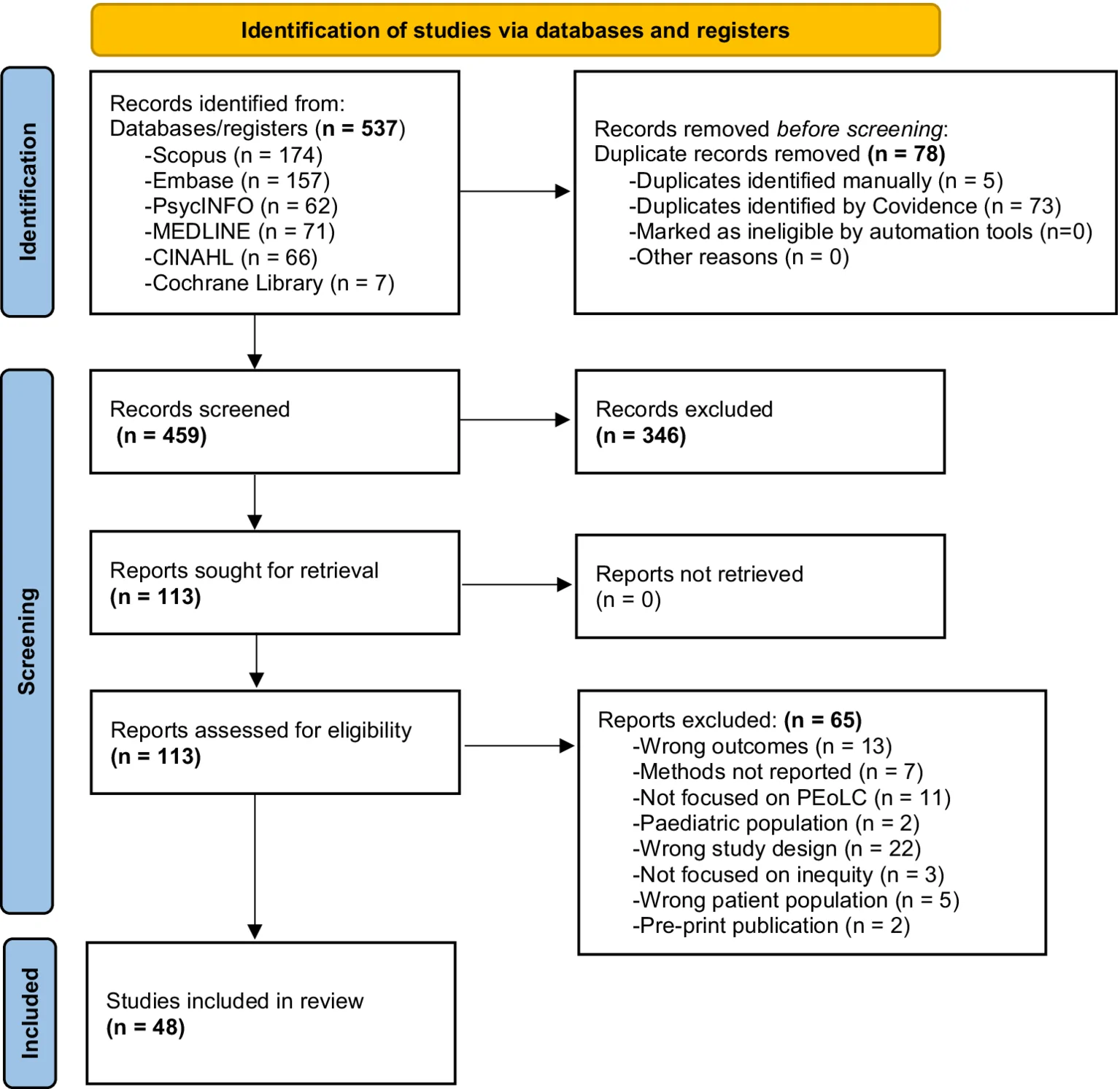
PRISMA flow diagram.

### Risk of bias

The risk of bias in the included reviews was assessed using the Joanna Briggs Institute’s Critical Appraisal Checklist for Systematic Reviews and Research Syntheses in order to transparently appraise quality and inform synthesis (18). Reviews were not excluded on the basis on the critical appraisal. This checklist comprises 11 questions addressing the review’s methodology and potential bias, with responses recorded as yes, no, unclear, or not applicable. The included reviews were divided among team members who independently assessed the quality of the assigned reviews. The assessments were independently verified by AP and MS. Any conflicts between reviewers were resolved through discussion. Risk of bias was undertaken No reviews were.

### Data synthesis

Following the data extraction process, the extracted data were exported as an Excel spreadsheet from the Covidence software. Data synthesis was undertaken by MS, CB, and AP. Microsoft Excel was used to tabulate the findings and generate statistics and figures. Findings regarding determinants of inequity, barriers and facilitators to accessing and palliative care and bereavement support and recommendations for improving equity, were categorised thematically and presented narratively.

## Results

A total of 537 records were identified through the initial and updates search (Figure 1). After removing duplicates, 459 records remained for title and abstract screening. Of these, 346 were irrelevant and excluded during this stage. The remaining 113 records were assessed for eligibility, and 65 were excluded based on the inclusion and exclusion criteria. A total of 48 reviews were included in the umbrella review.

### Characteristics of included reviews

The quality ratings of each review are presented in Supplementary File. The overall quality was good, and the methodological quality of the reviews was mostly strong. Four reviews stated an overall aim but did not provide a specific review question (19–22). The assessment of publication bias in the studies was unclear or unreported for most of the reviews.

Table 2 presents the characteristics of the included reviews (more detail in Supplementary File). The primary authors of the 48 reviews were from 10 different countries, most commonly the United States (*n* = 18, 37.5%) (19, 21, 23–38) and the UK (*n* = 16, 33.3%) (20, 39–53). The majority originated from high-income countries (*n* = 42, 87.5%) (19–30, 32–49, 52–63). While 27 of the 48 reviews (56.2%) included studies from multiple continents (20, 22, 27, 39–41, 43, 44, 46, 47, 49–54, 56–66), reviews focusing solely on populations from North America were the second most common comprising 19 reviews (39.5%) (19, 21, 23–26, 28–30, 32–38, 42, 48, 55). Only one review focused exclusively on participants from Europe (the UK and other European countries) (45).

**Table 2:** Details of included reviews.

Summarised reviews’ characteristics. (n) represents the number of papers with the characteristic		Total (*n* = 48)
Country in which the review conducted (based on the primary author affiliation)	United States, *n* (%)	18 (37.5)
	United Kingdom, *n* (%)	16 (33.3)
	Canada, *n* (%)	4 (8.3)
	Australia, *n* (%)	3 (6.2)
	Portugal, *n* (%)	2 (4.1)
	Denmark, *n* (%)	1 (2.0)
	Ecuador, *n* (%)	1 (2.0)
	New Zealand, *n* (%)	1 (2.0)
	Spain, *n* (%)	1 (2.0)
	South Africa, *n* (%)	1 (2.0)
Income of countries of primary studies	High, n *(%)*	42 (87.5)
	High and Middle, *n* (%)	2 (4.1)
	High and Low, *n* (%)	1 (2.0)
	Middle and Low, *n* (%)	1 (2.0)
	High; Middle and Low, *n* (%)	2 (4.1)
Country and continent (s) of origin of included primary studies	Mixed (more than one continent), *n* (%)	27 (56.2)
	North America only, *n* (%)	19 (39.5)
	South America only, *n* (%)	1 (2.0)
	Europe (United Kingdom and European Countries only), *n* (%)	1 (2.0)
Date of publication	2012–2015, *n* (%)	2 (4.1)
	2016–2019, *n* (%*)*	8 (16.6)
	2020–2023, *n* (%)	24 (50.0)
	2024–2025, *n* (%)	14 (29.1)
Type of review	Systematic review (as reported by the authors of the review), *n* (%)	11 (22.9)
	Mixed-methods systematic review, *n* (%)	1 (2.0)
	Systematic review with narrative synthesis, *n* (%)	3 (6.2)
	Scoping review, *n* (%)	19 (39.5)
	Integrative review, *n* (%)	7 (14.5)
	Qualitative synthesis, *n* (%)	4 (8.3)
	Rapid systematic review, *n* (%)	2 (4.1)
	Narrative review, *n* (%)	1 (2.0)
Population group(s)^$^ (which is considered in the studies based on equity)	Racialised communities, *n* (%)	25 (52.0)
	People experiencing economic deprivation, *n* (%)	6 (12.5)
	People with learning disabilities, *n* (%)	3 (6.2)
	People with mental health illness, *n* (%)	5 (10.4)
	People who are gender and /or sexually diverse groups, *n* (%)	9 (18.7)
	Older people, frailty and dementia, *n* (%)	8 (16.6)
	People experiencing homelessness, *n* (%)	4 (8.3)
	People with non-malignant disease, *n* (%)	5 (10.4)
	Remote or rural populations, *n* (%)	4 (8.3)
	Incarcerated or marginalise populations, *n* (%)	2 (4.1)
	Language justice and health literacy, *n* (%)	2 (4.1)
	People experiencing socio-economic deprivation, *n* (%)	5 (10.4)
	Religious minorities, *n* (%)	1 (2.0)
	People particularly in the low- and middle-income countries, *n* (%)	1 (2.0)
Extent to which primary studies have involved populations groups in research have directly engaged with the affected population groups.	No granular data, *n* (%)	26 (54.1)
	Population group; caregivers and families; health and social-care professionals, *n* (%)	9 (18.7)
	Population group only, *n* (%)	7 (14.5)
	Population group; healthcare professionals, *n* (%)	3 (6.2)
	Population group, caregivers and families, *n* (%)	3 (6.2)
Focus	Palliative, and end of life*, *n* (%)	21 (43.7)
	End of life only, *n* (%)	10 (20.8)
	Palliative only**, *n* (%)	9 (18.7)
	Palliative, end of life and advance care planning, *n* (%)	5 (10.4)
	Palliative, end of life and bereavement, *n* (%)	2 (4.1)
	Bereavement only, *n* (%)	1 (2.0)
Number of primary studies included	<10, *n* (%)	10 (20.8)
	11–15, *n* (%)	6 (12.5)
	16–20, *n* (%)	7 (14.5)
	21–25, *n* (%)	7 (14.5)
	26–30, *n* (%)	3 (6.2)
	31–35, *n* (%)	5 (10.4)
	35–40, *n* (%)	2 (4.1)
	41–45, *n* (%)	1 (2.0)
	51–60, n *(%)*	3 (6.2)
	>61, *n* (%)	4 (8.3)
Types of primary studies included	All study designs, *n* (%)	21 (43.7)
	Quantitative; Qualitative & Mixed methods research, *n* (%)	6 (12.5)
	Mixed methods only, *n* (%)	1 (2.0)
	Quantitative & Qualitative research, *n* (%)	4 (8.3)
	Quantitative research only, *n* (%)	11 (22.9)
	Qualitative research only, *n* (%)	5 (10.4)

Some reviews include more than one population groups such as racialised communities and older and frail individuals.

*Including hospice and intensive care units.

**Including palliative rehabilitation.

Half of the reviews were published between 2020 and 2023 (19–22, 27, 28, 30, 32, 33, 35–39, 43, 45, 46, 49, 50, 53, 59, 63, 65, 66), and 14 (29.1%) between 2024 and 2025 (23–25, 39, 41, 42, 54–58, 64). The predominant review type was scoping reviews (*n* = 19, 39.5%) (19, 22–26, 33, 35, 38, 40, 51, 53–55, 57–59, 62, 63) with various forms of systematic review (*n* = 17, 33.3%) (20, 28, 29, 34, 36, 37, 41, 42, 44, 46, 48, 50, 52, 56, 60, 65, 66).

Twenty-five reviews (52.0%) focused on racialised communities (19, 23, 24, 26, 28–31, 33–40, 42, 46, 48, 50, 51, 54, 55, 58, 62, 64). Nine reviews (18.7%) addressed the experiences of gender and/or sexually diverse groups (32, 38, 57, 58, 60–62, 64, 65), and eight reviews (16.6%) focused on older adults (37, 40, 44, 48, 53, 62, 64, 65) including those living with frailty and dementia. Definitions of the populations studied varied, with some reviews including more than one population group (23, 38, 40, 51, 58, 62–65). Twenty-one (43.7%) reviews examined both palliative and end-of-life care (19, 23, 24, 27, 34, 36, 39–41, 43, 45, 47, 48, 53–58, 61, 66), and 10 (20.8%) reviews focused exclusively on end-of-life care (26, 29, 31, 32, 37, 44, 49, 51, 52, 60). Only one review specifically addressed bereavement support (62).

#### Engagement with population groups experiencing inequity

Based on the reported findings within the reviews, direct engagement with population groups experiencing inequity in palliative care and bereavement support in the primary studies was inconsistent, often limited and heterogeneous. While some reviews included participants from the population groups identified as their sample (38, 49, 52, 65), the majority relied on proxy accounts from caregivers, family members, or healthcare professionals (20, 21, 26, 39, 44, 47, 48, 50, 53, 56, 59, 66). Although some studies directly included members of the population groups as participants, they were frequently included as a small subgroup within broader samples (20, 53, 55). For example, in a review, only 1% of the total sample were people with intellectual disabilities, and 1.3% were their family members, while the remainder were health and social care professionals (20).

The lack of centring on the perspectives of these population groups is a limitation. Most existing research has captured the needs and experiences of populations facing inequity indirectly, rather than through self-reported, meaningful, first-hand engagement. This leaves significant gaps in understanding and responding to their lived realities.

#### Barriers and facilitators contributing to inequity

The barriers and facilitators contributing to inequity, reported across the 48 reviews included in this umbrella review, are detailed in Table 3. They were categorised under three levels: individual, population and system-wide, while also recognising their interconnections. Key findings are described below.

**Table 3:** Barriers and facilitators contributing to inequity in palliative care and bereavement support across population groups.

Contributors to inequity/Barriers in palliative care and bereavement support		Mitigators to inequity/Facilitators in palliative, end of life and bereavement care
Barriers that are personal or interpersonal, often patient or caregiver-specific that may hinder equitable access to palliative care and bereavement support for individuals.	Individual	Facilitators related to personal behaviours, attitudes, and direct care delivery
Restricted communication (e.g., due to language, cognition) (20, 25–28, 31, 33–36, 40, 42–46, 48, 50, 51, 53, 54, 58, 59, 64, 66) Complexity of palliative, end-of-life and/or bereavement needs (28, 33, 35, 36, 39, 42, 44, 47, 51–54, 58, 61) Staff hesitancy/reluctance to care for dying people in population group (20, 41, 44, 45, 47, 58) Patients-related factors–poor health literacy, neurobiological differences, comorbidities (20, 34–36, 38, 47, 58) Poor communication between providers and patients/families (21, 35, 36, 44, 47, 51, 53, 56, 59) Uncertainty about what advanced care planning is (44, 64) Lack of understanding of service options (46, 55) Lack of trust in the services (36, 40, 58) Difficulties of identification of patients who is dying (20, 35, 47, 52, 58) Normalisation of dying (35, 47, 58)		Use of interpreters or other communication aids (21, 25, 26, 58, 67) A conversation “game” increased discussion and advanced care planning completion (19, 25) Increased staff compassion (20, 56, 58) Improved integration of service users (20, 39, 56) Understanding complexity and vulnerability (62) Improved person care including advanced care planning (20, 25–28, 33, 35, 37, 39, 41, 43, 47, 54, 59, 61, 63) Being respectful to patients (47) Skilled observation tools (e.g., DisDAT, PALLI) (20)
Barriers that reflect social, cultural and group-level challenges faced by specific groups experiencing inequity such as those associated with particular cultural, demographic, or socioeconomic characteristics.	Population-based	Facilitator related to group-level, cultural, community-oriented, and family-inclusive strategies
Poor awareness of palliative care services within population group (19–21, 23, 28, 34–36, 43, 45–47, 51, 52, 54, 55, 58, 62, 63) Lack of shared decision-making with patient and family healthcare staff (25, 36, 40, 43, 58) Lack of grief support for both individuals and staff (56) Lack of family involvement in population group (19, 20, 27, 34–36, 43, 44, 49, 54, 56–58, 61) Late diagnosis and/or recognition of disease progression and/or dying by healthcare staff (20, 35, 36, 43, 45, 47, 52, 58) Stigmatisation of population group (27, 32, 40, 41, 45, 46, 57, 61, 62, 65, 66) Cultural barriers (19–21, 24–26, 28, 30, 31, 33–38, 42, 45, 46, 48, 50, 51, 53–55, 58, 66) Racial discrimination (25, 33–36, 38, 46, 51, 58, 65) Religious belief (24, 35, 40) Low income and financial constraints (22, 36, 46, 47, 49, 51, 55, 58)		Educational resources for population (19, 20, 26, 28, 31, 32, 34, 40, 55) Feedback from patients about the barriers they face in receiving palliative care (22) Better understanding and communication (25, 47, 51, 59) Educational resources for family (19, 20, 26, 40, 55, 58) Improved family involvement in care (20, 30–32, 34, 40, 43, 48, 54, 55, 58, 65) Patient navigator and tailored interventions (34) Improving trust in healthcare (33, 47, 64) Awareness, understanding, and respecting cultural beliefs and values (25, 35, 51, 61) Culturally respectful advanced care planning information (25, 35, 40, 55, 58, 64) Translating medical terminology into simpler concepts (20) Need for intervention specific to population and their families (19)
Barriers related to services, infrastructure, policy and healthcare systems -systemic and structural inequities within the healthcare system and broader society	System-wide	Facilitators related to infrastructure, policy, education/training systems, and multidisciplinary models
Inadequate staff expertise regarding how to care for population group (20, 21, 27, 30–32, 35, 39, 41, 43, 45, 47, 50, 53, 54, 57–59, 61, 63, 66) Lack of tailored provision for population group (26, 27, 31–35, 39, 42, 43, 45–47, 51, 54–56, 58, 59, 63) Absence of relevant guidelines, policies and protocol for population group (19–21, 23, 39, 43, 51, 58, 59, 63) Poor service provision for population group (20, 23, 27, 33, 35–37, 39, 40, 42, 43, 46, 47, 53, 54, 56, 58, 61, 63) Under referral to palliative care (21, 35, 36, 47, 53, 59) Lack of supportive legislation from government (21, 35, 36, 53) Professional and time limitations–Administrative barriers (44, 58) Lack of care to facilitate care in preferred place (20, 33, 35, 36, 38, 41, 49, 56, 58, 65) Limited experience and preparation of the healthcare providers (47, 59) Environmental barriers (e.g., shackles, lack of physical/other comforts) (35, 47, 51, 53, 56) Inconsistencies across palliative care research specific to intervention development with minoritised populations with serious illness (19, 23, 35, 38, 47, 51) Lack of risk and safety assessment (58) Lack of funding (21) Rural and remote areas (38, 46, 51, 59) Lack of advocacy (41)		Education re population group included in undergraduate education (19, 40, 45, 59) Specialist postgraduate education on care for population group (21, 24, 58) Educational resource kits for staff (20, 27, 30, 35, 40, 42, 50, 56, 58, 63) Improvements to multidisciplinary/multiagency care (19, 20, 33, 35, 41–43, 47, 50, 58, 63, 65) Interdisciplinary training in communication (29, 33) Caregiving is a policy concern (60) Liaison and/or link staff (e.g., trained link nurses) (20, 35, 41, 45, 56) Public health approach (20, 23, 55, 58, 65) Earlier involvement with specialist palliative care (20) Require instruction on basic palliative care principles, pain management, planning, hospice care options (27, 59) More training and qualification in the care of specific physical, spiritual, social, and emotional needs (56, 59) Increased staffing (53) Respecting Choices model (61, 64) Upskilling of general workforce (19, 20, 58) Increased awareness of palliative and end of life care (54, 58) Community-based participatory research (23, 58) Development or use of specialist assessment tools (20, 63) Establishing communication links with local hospices (56, 58) Community outreach and services (19, 20, 33, 47, 58) Emotional support for staff (20)

### Individual barriers and facilitators

Individual related barriers refer to the unique personal, family and staff factors that may hinder equitable access to palliative care and bereavement support. Restricted communication (e.g., due to language barriers or cognitive impairment) (20, 25–28, 31, 33–36, 40, 42–46, 48, 50, 51, 53, 54, 58, 59, 64, 66) and poor understanding or awareness of available services (46, 55) fell within this category. Other identified person-related factors include low health literacy (20, 34–36, 38, 47, 58), complex health needs (28, 33, 35, 36, 39, 42, 44, 47, 51–54, 58, 61), the normalisation of dying (35, 47, 58) and uncertainty regarding disease progression and care needs (44, 64). Staff hesitation or reluctance to provide care for dying people within certain population groups also increased the risk of inequity in palliative care (20, 41, 44, 45, 47, 58).

Facilitators related to individual behaviours, attitudes and direct care delivery included the use of interpreters and other communication aids (21, 25, 26, 58, 67); showing respect towards patients (47); improving integration of service users into care planning and delivery (20, 39, 56); and maintaining a strong focus on person-centred care (20, 25–28, 33, 35, 37, 39, 41, 43, 47, 54, 59, 61, 63).

### Population-based barriers and facilitators

Population-based barriers refer to the challenges faced by specific groups experiencing inequity often linked to cultural, demographic, or socioeconomic characteristics. These included: poor awareness and engagement of the population group and their families (19–21, 23, 27, 28, 34–36, 43–47, 49, 51, 52, 54–58, 61–63); a lack of shared decision-making involving patients, families, and healthcare providers (25, 36, 40, 43, 58); stigmatisation of the population group within health and social care (27, 32, 40, 41, 45, 46, 57, 61, 62, 65, 66); late diagnosis and delayed recognition of dying within the group (20, 35, 36, 43, 45, 47, 52, 58); and socioeconomic or other individual factors (such as cultural differences (19–21, 24–26, 28, 30, 31, 33–38, 42, 45, 46, 48, 50, 51, 53–55, 58, 66), racial discrimination (25, 33–36, 38, 46, 51, 58, 65), religious beliefs (24, 35, 40), and low income and financial constraints (22, 36, 46, 47, 49, 51, 55, 58).

Population-based facilitators included: delivering more culturally and community-oriented care (by understanding cultural beliefs and values and providing culturally respectful care and information) (25, 35, 40, 51, 55, 58, 61, 64); improving education and communication between populations, their families and healthcare teams through accessible resources (19, 20, 26, 28, 31, 32, 34, 40, 55, 58), enhancing public understanding of medical terminology (20); and strengthening family (20, 30–32, 34, 40, 43, 48, 54, 55, 58, 65) and population engagement to build greater trust in healthcare (33, 47, 64).

### System-wide barriers and facilitators

System-wide barriers reflect inequities embedded within healthcare systems and the wider society. These include issues related to service provision, infrastructure, policy, and governance, which collectively hinder equitable access to palliative care and bereavement support. Those identified within the reviews were: a lack of workforce knowledge and capacity (20, 21, 27, 30–32, 35, 39, 41, 43, 45, 47, 50, 53, 54, 57–59, 61, 63, 66), professional and time limitations (44, 58); service gaps and lack of accessibility (20, 23, 27, 33, 35–37, 39, 40, 42, 43, 46, 47, 53, 54, 56, 58, 61, 63), under-referral to palliative care (21, 35, 36, 47, 53, 59), challenges associated with living at rural and remote areas (38, 46, 51, 59); the absence of appropriate policy, guidelines, and funding (19–21, 23, 39, 43, 51, 58, 59, 63), as well as inadequate funding (21) and advocacy (41).

System-wide facilitators, including infrastructure, policy, education and training systems, and multidisciplinary models were reported as potentially helping to reduce inequities in and palliative care and bereavement support. There is a clear need for service and workforce development, including improvements to multidisciplinary and multi-agency care (19, 20, 33, 35, 41–43, 47, 50, 58, 63, 65), increasing staffing levels, and establishing effective communication links with local services (56, 58). Other facilitators included the provision of well-established education and training to enhance care delivery and communication with specific population groups (19, 20, 27, 29, 30, 33, 35, 40, 42, 45, 50, 56, 58, 59, 63); the development of public- and population-based approaches and models of care (20, 23, 55, 58, 65); the provision of emotional support for staff (20); policy enhancements to strengthen caregiving and carer roles (60); and the creation of specific assessment tools and educational resources for staff (20, 63).

#### Structural determinants contributing to inequity across population groups

As shown in Figure 2, various structural determinants of inequity span population groups. Firstly, low economic status impedes access to services due to financial constraints, including lack of insurance and income, inability to afford healthcare, loss of income, poor housing, homelessness, and poverty (21, 23, 27, 40, 41, 45–47, 49, 51, 54, 55, 58, 61, 63, 66). Personal factors such as poor health literacy and limited understanding and awareness of care and service provision also span population groups experiencing inequity (21, 34, 40, 41, 45, 51, 55, 58, 63, 64). Cultural and language dissonance including cultural barriers (19, 21, 23, 26, 28, 30, 31, 33–37, 42, 45, 48, 50, 51, 53–55, 58, 64–66), language difficulties (23, 25, 26, 28, 31, 34, 36, 40, 42, 45, 46, 51, 53–55, 58, 59, 64), and socio-cultural expectations (60, 66) - are further structural determinants that were found to increase the likelihood of experiencing inequity. Geographical factors are associated with inequities in accessing care because they influence the availability of services in individuals’ locations (21, 23, 38, 45, 46, 51, 53, 55, 56, 58, 59, 66). Informal caregiver discrepancies were also included in this group (58). Finally, vulnerability related to personal and clinical characteristics is highlighted. Factors such as belonging to a minoritised ethnic group, age, and gender identity contribute to vulnerability, including both physical and psychological aspects (21, 23, 27, 35, 44, 46, 51, 52, 57–59, 61, 62, 65). These include social exclusion, social isolation and feelings of exclusion due to stigma associated with their group identity or age (26, 27, 32, 36, 43, 53, 58, 61).

**Figure 2:**
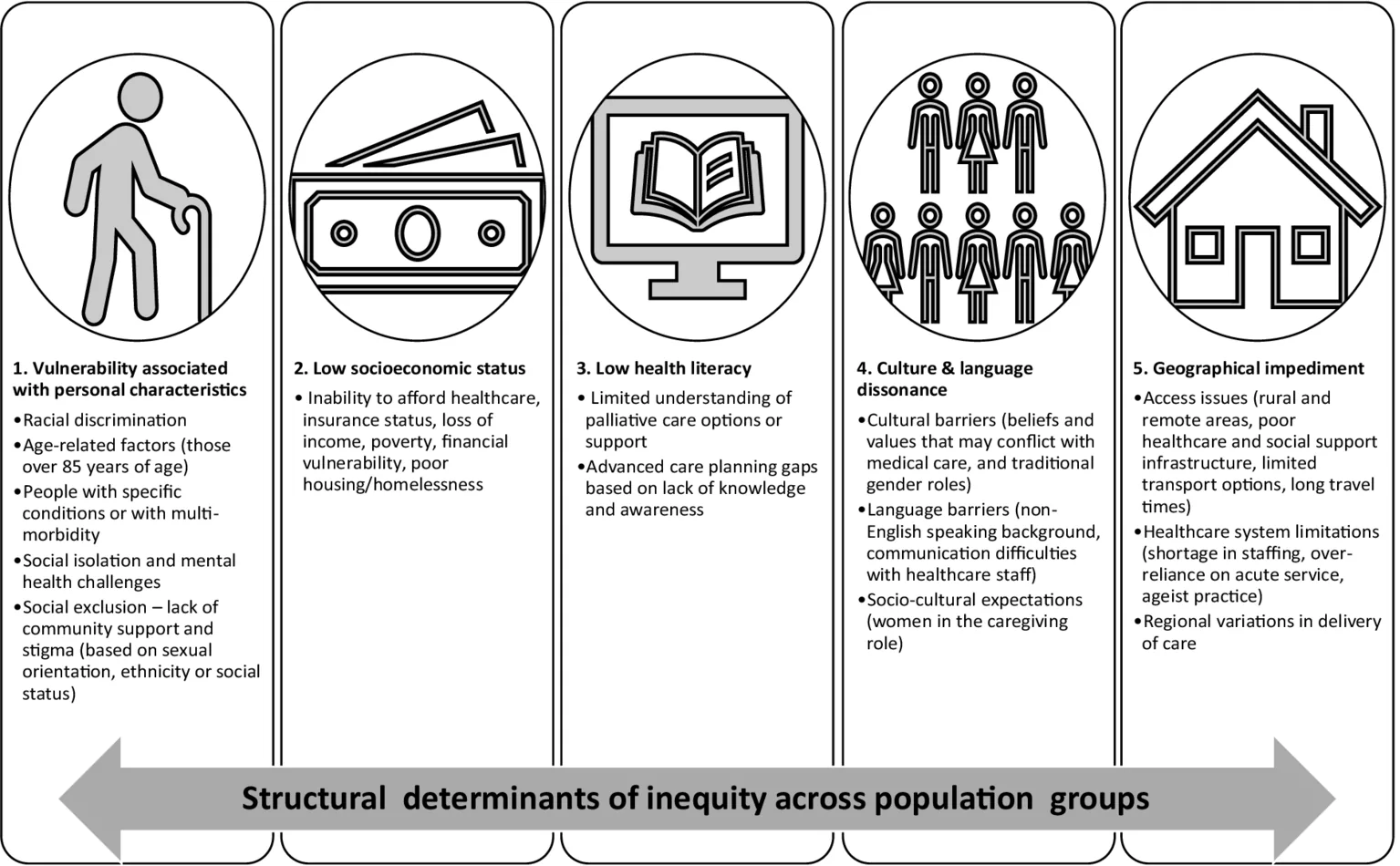
Structural determinants contributing to inequity across population groups.

#### Existing recommendations to reduce inequity

The recommendations for education and training, policy, practice, and future research identified within the reviews are highlighted in Table 4.

**Table 4:** Recommendation for reducing inequity in palliative and end of life care.

Recommendations	
Education	Cultural competence and intersectionality (25, 26, 29, 32, 36, 37, 39, 41, 42, 50, 54, 55, 57, 58, 64) Train healthcare staff in cultural competence, inclusive communication, and intersectional awareness. Educate on cultural, historical, and social influences in end-of-life care. Provide cross-cultural communication training for tailored interventions. Embed gender-inclusive and spiritually sensitive care, with safe spaces and support. Standardise culturally competent advance care planning training. Integrate equity and ethnicity content in health curricula. Support effective interpreter use, including training and emotional support. Include race-informed approaches in teaching goals-of-care conversations.
	Professional education and curriculum development (19, 25, 26, 32, 37, 41–43, 47, 56, 59, 60, 63, 66) Integrate palliative care into all training levels—from carers to clinicians to specialist professionals. Promote ongoing training on equitable care and recognising structural racism. Train clinical teams (e.g., stroke teams) in shared decision-making. Build trauma-informed, cross-sector education into emergency planning. Provide interdisciplinary training for effective end-of-life communication. Ensure inclusion of content on intellectual disability and palliative care in medical and nursing education. Train clinicians in working with interpreters Address system-wide barriers in education and communication through policy and standardised guidance.
	Healthcare staff support (41, 44, 47, 63) Offer training and emotional support for staff managing complex emotional and practical care demands. Provide specialist training and support for those working with people experiencing homelessness. Ensure the workforce is adequately trained and supported.
	Education for patients, families, carers, and public (19, 29, 34, 54, 55, 59) Promote public engagement and education on palliative care to increase public awareness of palliative care and its benefits. Support community and family involvement in care Provide training for family carers and in-home support staff, enabling respite and sustainable caregiving. Improve patient education and understanding of care options.
	System navigation and equity (20, 36, 42, 58) Offer support to navigate healthcare systems, especially for marginalised groups. Train providers to address intersectional needs. Ensure providers are trained to recognise and respond to intersectional needs. Tackle education and communication barriers in palliative and end-of-life care access. Use targeted social work interventions to support family involvement and post-ICU care planning.
Policy (priorities)	Language and communication (20, 24, 26, 29, 44, 54, 57–59) Promote linguistically sensitive, inclusive care through language justice, interpretation services, and standardised terminology. Standardise definitions of palliative and end-of-life care to avoid fragmented understanding. Address system-wide barriers in education and communication through policy and standardised guidance. Preserve the autonomy of the individuals
	Cultural safety and equity (19, 22, 26, 29, 32, 42, 45, 47, 50, 51, 54, 56, 58, 59, 63–65) Ensure culturally competent, inclusive and anti-discriminatory policies that respect treatment preferences from the earliest stages. Address intersectionality (e.g., gender, ethnicity, spirituality) and avoid one-size-fits-all approaches. Awareness of personal own biases and address them to better support patents and challenge gendered expectations
	System integration and approaches (19, 21, 29, 42, 46, 53, 58, 60) Embed equity across health and social care, not just hospices with a recognition of the impact of social determinants Expand community-based models and tackle systemic barriers (e.g., insurance, geography, cost).
	Advocacy and accountability (26, 29, 49, 50, 56, 58) Involve patients, families, and communities in policymaking to strengthen the frameworks to support equitable access and quality of care Support trauma-informed, flexible, and person-centred policies for marginalised groups.
	Decision-making and person-centredness (26, 29, 31, 51, 58) Develop culturally respectful frameworks for life-sustaining decisions, family-centred planning, and clear palliative care guidelines in primary care.
	Evidence-based and data-informed policy (20, 26, 46, 58, 59, 63) Use culturally tailored strategies, disaggregated data, and context-specific definitions of disadvantage to inform equitable policy. Implement evidence-based, community-informed recommendations.
	Caregiver and workforce support (35, 58) Invest in training, protection, and support for informal caregivers and long-term care staff, especially from underrepresented and gender-diverse groups. Address policy-driven inequities in caregiving burdens
Practice	Culturally safe, inclusive care and effective communication (20, 24, 26–28, 31, 32, 34–37, 40, 42, 43, 45, 50, 54, 56, 58, 59, 61–63, 65) Provide culturally tailored, bias-free training for healthcare staff. Include non-traditional family structures in care planning. Use bilingual and faith-based care education materials for underserved groups. Respect cultural values, (religious or spiritual) beliefs, and communication preferences. Involve human interpreters or cultural mediators for accurate, sensitive communication. Assistive technologies use to improve communication when requires
	Building trust and relationships (30, 35, 39, 45, 47) Partner with trusted community figures (e.g., church leaders, Traveller advocates). Ensure continuity of care, especially for marginalised groups (e.g., those with mental health illnesses, homeless individuals). Apply community-based participatory approaches in service design and delivery.
	Providing equitable access (21, 36, 37, 40, 42, 43, 45, 46, 52, 55, 57, 63, 65, 66) Adapt and extend palliative and end of life care for underserved groups (e.g., Black communities, LGBT+, prisoners, Travellers) and their carers. Awareness of the severity of conidiation and palliative care access/preferences Expand outreach and telehealth in rural and deprived areas. Supporting carers
	Informed shared decision-making and person-centred care (and education) (19, 20, 28, 40, 43–45, 48, 55, 56, 58, 61, 64, 65) Start palliative and end of life care initiatives early with honest, compassionate communication. Respect the right to decline; avoid coercion. Embed advanced care initiatives into routine care with protocols and regular reviews. Train staff in shared decision-making, especially in uncertain situations. Offer personalised support and adapt care to setting, age, gender, and condition.
	Intersectionality and tailored support and intervention (26, 28, 32, 34–36, 41, 42, 44, 47, 49, 53, 60) Address overlapping barriers (e.g., race, gender, disability, housing). Design inclusive, community-informed interventions. Provide a multi-faced approach to address broad ranging and complex issues Promote gender-sensitive care (e.g., women’s mental health, male stoicism).
	System-level improvements (20, 27, 43, 45, 62, 63) Strengthen collaboration between services (e.g., GPs, stroke units, palliative care teams). Tackle structural issues like under-resourcing and fragmented bereavement care. Ensure adequate staffing, training, and staff wellbeing.
	Education and advocacy (21, 30, 35, 44, 45, 50, 59–61) Provide culturally appropriate, bilingual education to support advanced care planning uptake. Build clinician confidence in end-of-life conversations. Support advocacy by training peer mentors, faith leaders, and frontline staff. Provide education for patients, family members and caregivers
Research	Equity and inclusion in underserved populations (19, 20, 24, 26–28, 31–37, 42, 45–48, 50, 54, 55, 57, 61–63) *African, black and minority ethnic groups* Explore intergenerational experiences of African migrants. Investigate the effects of racism, structural vulnerabilities, and social determinants on palliative and end-of-life care. Improve recruitment and ensure culturally appropriate research. *LGBTQIA+ communities* Conduct participatory, intersectional research on needs and experiences of LGBTQIA+ individuals and carers. Develop and evaluate inclusive, identity-affirming palliative and end-of-life care models and training. *Travelling and homeless communities* Co-produce research with travelling and itinerant groups. Examine palliative and end-of-life care access for homeless populations, especially women and those with severe mental illness. Consider housing history and social context in care research. *People with intellectual disabilities* Assess tools (e.g., DisDAT), training, and link nurse models. Develop validated outcomes tailored to this population. *People who use drugs* Expand palliative and end-of-life care research beyond HIV/AIDS to broader substance use, especially during health crises.
	Intersectionality and structural inequities (19–22, 24, 28, 31–33, 37, 40–42, 44, 45, 47–51, 58, 59, 61, 62) Apply intersectional approaches (e.g., race, gender, geography, socioeconomic status) to understand overlapping disadvantages Investigate systemic barriers and design equity-focused interventions. Address rushed, impersonal care as a barrier to holistic palliative and end-of-life care.
	Community-based and culturally appropriate research (21–25, 28, 30, 34, 35, 45, 51, 54, 57, 62) Use community-based participatory research and co-production with underrepresented groups. Test and adapt culturally tailored interventions. Engage interpreters, community advisors, and cultural navigators in study design. Build trust with communities with histories of marginalisation (e.g., Black faith groups). Include spiritual, social, and cultural aspects of care in what matters to patients or needs in patient priorities
	Models of care and interventions (20, 23–26, 28, 33, 35, 41–45, 51–53, 56, 57, 59) Develop equity-informed, system-level models and peer support strategies. Develop language justice and peer navigation strategies Community-based and multicomponent interventions (e.g., RCTs using socio-ecological frameworks) Evaluate symptom management (e.g., pain, delirium, psychological distress), early referrals, and patient-centred outcomes including quality of life). Develop (multidimensional) tools for older adults with multimorbidity.
	Methodological approaches (21, 24, 28, 33, 35, 38, 42, 43, 45, 48, 51, 59, 61) Use mixed-methods, longitudinal studies and implementation science - combine qualitative and quantitative data to understand access and quality of care. Standardise measures and outcomes, define ethnicity appropriately, and ensure diverse, cross-national recruitment (e.g., younger adults, rural, men, non-cancer patients). Design national surveys using culturally valid tools.
	Disease- and population-specific needs (20, 26–28, 33, 35, 36, 38, 42, 43, 45, 48, 52, 64) Explore barriers/facilitators to advance care planning. Understand low uptake of advance directives and develop inclusive approaches. Identify palliative and end-of-life care disparities across diseases (e.g., heart failure, chronic obstructive pulmonary disease, stroke, chronic kidney disease, dementia, diabetes, HIV/AIDS) Examine care transitions, symptom burden, family roles, and care experiences in acute/intensive care unit settings.
	Workforce and training (20, 25, 26, 35, 40, 45, 48, 57, 59, 60) Explore professional and carer experiences with underserved groups. Evaluate training in cultural competence, communication, and pain/symptom management. Develop roles for liaison staff, peer educators, interpreters, and support workers.

### Recommendations for education and training

The included reviews recommended that the training of healthcare providers should encompass cultural competence, inclusive communication, and intersectional awareness in care, alongside the provision of ongoing education and emotional support (25, 26, 29, 32, 36, 37, 39, 41, 42, 50, 54, 55, 57, 58, 64). Professional education should integrate palliative care into all undergraduate curricula (19, 25, 26, 32, 37, 41–43, 47, 56, 59, 60, 63, 66). For patients, families, and the wider public, the recommendations included promoting public engagement and education on palliative care to increase awareness of its benefits, and improving patient education to enhance understanding of care options (19, 29, 34, 54, 55, 59). To improve system navigation and equity (20, 36, 42, 58), the reviews suggested offering tailored support to help individuals navigate healthcare systems, particularly for marginalised groups; addressing educational and communication barriers in accessing palliative care; and employing targeted social work interventions to support family involvement.

### Recommendations for policy

The following policy recommendations were identified to improve equity in palliative care: promote linguistically sensitive and inclusive care (20, 24, 26, 29, 44, 54, 57–59) through language justice, interpretation services, and the use of standardised terminology; ensure that policies are culturally competent (19, 22, 26, 29, 32, 42, 45, 47, 50, 51, 54, 56, 58, 59, 63–65), inclusive, and anti-discriminatory and that they respect treatment preferences from the earliest stages of care; address intersectionality and avoid one-size-fits-all approaches; expand community-based models and address systemic barriers (19, 21, 29, 42, 46, 53, 58, 60); involve patients, families, and communities in policy making (26, 29, 49, 50, 56, 58) to strengthen frameworks that support equitable access and quality of care; use culturally tailored strategies, disaggregated data and context-specific definitions of disadvantage to inform equitable policy (20, 26, 46, 58, 59, 63); and invest in training, protection, and support for informal caregivers and long-term care staff (35, 58), particularly those from underrepresented and gender-diverse groups.

### Recommendations for practice

The reviews identified several practice-focused recommendations to improve equity in palliative care. These include providing culturally safe and inclusive care (20, 24, 26–28, 31, 32, 34–37, 40, 42, 43, 45, 50, 54, 56, 58, 59, 61–63, 65) by delivering culturally tailored, bias-free training for healthcare staff and respecting cultural values, beliefs, and communication preferences. Building trust and relationships (30, 35, 39, 45, 47) was highlighted as essential, with suggestions to partner with trusted community figures (e.g., church leaders, traveller advocates) and apply community-based participatory approaches in service design and delivery. Ensuring equitable access (21, 36, 37, 40, 42, 43, 45, 46, 52, 55, 57, 63, 65, 66) was recommended through adapting and extending palliative care for underserved groups and offering outreach and telehealth services in rural and socioeconomically deprived areas. Facilitating shared decision-making and person-centred care (19, 20, 28, 40, 43–45, 48, 55, 56, 58, 61, 64, 65) was advised by training staff in shared decision-making, particularly in situations of uncertainty, and providing personalised support tailored to setting, age, gender, and condition. Addressing intersectionality and providing tailored interventions (26, 28, 32, 34–36, 41, 42, 44, 47, 49, 53, 60) was also emphasised, with a focus on tackling overlapping barriers and designing inclusive, community-informed interventions. System-level improvements (20, 27, 43, 45, 62, 63) were recommended, including strengthening collaboration between relevant services. Finally, promoting education and advocacy (21, 30, 35, 44, 45, 50, 59–61) was encouraged through training peer mentors, faith leaders, and frontline staff to support awareness and engagement in palliative care.

### Recommendations for research

Inclusion of underserved populations (including African, Black and minority ethnic groups, LGBTQIA+ communities, Travelling and homeless communities, people with intellectual disabilities, and people who use drugs) (19, 20, 24, 26–28, 31–37, 42, 45–48, 50, 54, 55, 57, 61–63) is essential to ensure culturally appropriate and inclusive research informed by the needs and experiences of these groups. Applying intersectional approaches can illuminate overlapping disadvantages and structural inequities (19–22, 24, 28, 31–33, 37, 40–42, 44, 45, 47–51, 58, 59, 61, 62). Research should prioritise community-based, culturally appropriate methods and co-production with underrepresented populations to test and adapt tailored interventions (21–25, 28, 30, 34, 35, 45, 51, 54, 57, 62). This includes developing equity-informed system-level models, peer support strategies, language justice initiatives, and peer navigation approaches (20, 23–26, 28, 33, 35, 41–45, 51–53, 56, 57, 59). Mixed-methods, longitudinal studies, and implementation science can combine qualitative and quantitative data to examine access and quality of care (21, 24, 28, 33, 35, 38, 42, 43, 45, 48, 51, 59, 61). Disease and population-specific research should explore barriers and facilitators to advance care planning and identify palliative care disparities across conditions such as heart failure, chronic obstructive pulmonary disease, stroke, chronic kidney disease, dementia, diabetes, and HIV/AIDS (20, 26–28, 33, 35, 36, 38, 42, 43, 45, 48, 52, 64). To strengthen the healthcare workforce, studies should explore professionals’ experiences with underserved groups, evaluate training in cultural competence, communication, and pain/symptom management, and develop roles for liaison staff, peer educators, interpreters, and support workers (20, 25, 26, 35, 40, 45, 48, 57, 59, 60).

## Discussion

### Summary of key findings

To the authors’ knowledge, this umbrella review is the first to have synthesised the reviews of research into population groups known to experience inequality in palliative and bereavement care. It has found such reviews are growing exponentially, are mostly undertaken in high income countries, consider palliative care and the population group of racialised communities. There is paucity of systematic reviews considering bereavement care. The population groups and markers of inequity including accessibility, need and positive outcomes are conceptually heterogenous. There are reviews on single populations such as those with learning disabilities (20), sub-groups of populations known to experience inequity such as African American migrants (54) and intersectional populations such as older adults within racialised communities (31).

Definitions are not standardised such that the constituents of population groups in the studies differ. For example, two systematic reviews explore the needs and experiences of ‘older people’ yet use different criteria for this population group when defining their scope. One which aimed to ‘investigate the attitudes of the public and healthcare professionals to advance care planning with frail and older people’ does not outline who classifies as ‘frail’ and ‘older’; the other which seeks to ‘understand the palliative care needs of community dwelling people aged >60 living with multi-morbidity in the last two years of life’ sets age criteria and defines multi-morbidity and frailty in line with national guidelines (53). Similarly, the experience of groups that are fluid, such as people who have historical experiences of homelessness or incarceration, has not been reviewed. The research relating to some identified population groups has not been systematically reviewed, for example people living rurally. Wider literature indicates there are groups of people who experience wider health inequity, such as sex-workers, whose experience of inequity in palliative care and bereavement support have not been explored (68).

The reviews predominantly use a scoping or systematic methodology to synthesise primary research describing inequities of needs and experiences of services, often with a broad scope. No reviews developed or evaluated interventions to reduce inequity. Data collected directly from people within the affected population groups is limited, with health professionals and family members serving as proxies in primary research and reviews considering those with cognitive impairment (20, 53). There is no evidence whether people with experience of inequity have been involved in review design.

This heterogeneity, limited involvement of people with lived experience and lack of solutions is challenging for service providers commissioners seeking to reduce inequity within across and beyond population groups known to experience it. UK health inequality reduction strategy acknowledges the structural, geographic and systemic equality that persists in palliative care and highlights the need for robust data to inform commissioning, policy and service development and evaluation (11, 69). Similarly, engagement with communities is key to inequity reduction. However, both strategies are difficult if groups are ill-defined and fluid, and research is not solutions focused nor comprehensive.

While some facilitators and barriers are unique to specific population, for example, workforce education on a populations’ specific needs, most facilitators and barriers transcend a range of groups, for example, restricted communication occurs in people with intellectual disability (20), mental health issues (26) and ethnic minorities (25). Recommendations from the reviews are similarly complementary: for example, service and workforce development (19, 20, 33, 35, 41–43, 47, 50, 58, 63, 65). These common structural determinants of inequity in palliative care and bereavement support reflect wider determinants of disadvantage healthcare which are often categorised as socio-economic, geographic, those relating to specific characteristics and social exclusion (70), all of which are evident within the research relating to palliative care and bereavement support. The findings suggest it is more useful to identify inequity through the determinants we have identified than through population groups alone.

### Implications for policy

The commonality across barriers, facilitations and structural determinants of inequities in population groups experiencing inequity mean that systematically addressing the common facilitators across groups, alongside focusing on the needs of unique needs of specific population groups where indicated may significantly impact on improving inclusive and personalised care for all regardless of the population group or groups to which they have or do belong. This inclusive public health approach also addresses the challenges of effectively delineating, identifying and encompassing all the groups. The Health and Social Care Committee’s Expert Panel: Evaluation of palliative care in England supports these findings (11). They emphasise that challenges do not exist in isolation and the inequalities in palliative care are interconnected. People who belong to multiple marginalised groups often face overlapping barriers, placing them at the greatest risk of poor care. The panel highlighted the need for further exploration into the persistent structural and systemic inequities in palliative care and the geographical inequalities in access and outcomes of palliative care.

### Implications for education

Education and training of healthcare staff, populations that experience inequity families and the wider public is a common recommendation of systematic reviews in the field. It has the potential to improve health literacy, understanding services within and across populations experiencing inequity and improve cultural competency within healthcare staff. However, robust evidence is needed to demonstrate the impact of education to reduce inequity.

### Implications for research

Where focusing on reducing inequality by focusing on the unique experiences of a particular group is warranted, future research develops conceptual consistency enabling useful synthesise of their unique experience. Research and reviews of research considering inequity within bereavement is needed to better understand this area. Research that develops and evaluates tools and interventions to reduce inequality within, across and beyond population groups is also warranted.

### Implications for practice

Palliative care and bereavement support service providers may usefully consider the scope and potential contribution of the speciality in addressing inequity both within their services and within wider healthcare. For example, hospices have strong community networks and increasingly public health-based initiative which may be harnessed to reduce structural determinants of inequity both within palliative care and bereavement support but also beyond it.

### Strengths and limitations

The umbrella review has synthesised international research across broad and heterogeneous fields. However, it relies on secondary analysis of published systematic reviews and carries inherent limitations related to their quality and heterogeneity. The terminology and conceptual descriptions of inequity/equity, palliative care and bereavement support are broad and varied. Similarly, descriptions of concepts of ‘need’ and ‘access’ to care and support are also heterogeneous across the systematic reviews. Consequently, population-based discrimination or inequity-related studies may have been overlooked despite the use of common search terms for these concepts. Findings must be interpreted with caution given the concepts may be understood or applied inconsistently across the systematic reviews. Additionally, the majority of the reviews were from high- and middle-income countries, which suggests a high likelihood that inequities experienced by people in low-income countries were underrepresented. This is particularly relevant given that many developing countries are in the early stages of palliative care service development. Considering system-wide barriers, it is highly probable that individuals in these settings face distinct challenges or lack adequate support. The majority of reviews focused on palliative and end-of-life care, with a notable lack of evidence regarding bereavement support for both underserved populations and their families. Only one review exclusively addressed this area. Based on the population groups considered in this review, the majority of the reviews focused on racialised communities, which may have influenced the findings to predominantly reflect the experiences of this group.

## Conclusion

This review highlighted that inequity in palliative care and bereavement support reflects collective and shared experiences across underserved population groups. It is therefore likely more effective to ensure care is tailored to the individual, irrespective of the population group to which they belong. Understanding the interconnected and intersectional nature of individual, population, and system-wide barriers and facilitators is essential. This finding should help prevent commissioning and service provision aimed at addressing inequity from being overly focused on one specific community, thereby benefiting wider groups experiencing inequity. Addressing these issues requires inclusive collaboration and the development of equity tools and interventions to improve care for all groups.
