# Supplementary material for: Inequity in palliative care and bereavement support: an umbrella review of the evidence
Source: Front Public Health. 2026 Oct 19;14:1943244. doi: 10.3389/fpubh.2026.1943244 (PMC13725816; doi:10.3389/fpubh.2026.1943244)
Supplement: Supplementary file 1 [file Data_Sheet_1.DOCX]

**Supplementary materials**

**1 Medline search syntax**

Database: Ovid MEDLINE(R) ALL <1946 to May 05, 2025>
Search Strategy:
--------------------------------------------------------------------------------
1     exp Palliative Care/ (67355)
2     exp Terminal Care/ (59764)
3     exp Bereavement/ (16210)
4     exp Hospices/ (6024)
5     ('end of life' or end-of life or Palliative* or Terminal or Hopsic* or Bereave*).ti,ab. (578778)
6     1 or 2 or 3 or 4 or 5 (643451)
7     (inequit* or unequit* or unequal* or inequal* or disadvant*).ti,ab. (198136)
8     6 and 7 (2262)
9     limit 8 to (yr="2012 -Current" and ("scoping review" or "systematic review")) (71)

***************************

**Table 2. JBI Critical Appraisal Checklist for included reviews**

| Study ID | Is the review question clearly and explicitly stated? | Were the inclusion criteria appropriate for the review question? | Was the search strategy appropriate? | Were the sources and resources used to search for the studies adequate? | Were the criteria for appraising studies appropriate? | Was critical appraisal conducted by two or more reviewers independently? | Were there methods to minimise errors in data extraction? | Were the methods used to combine studies appropriate? | Was the likelihood of publication bias assessed? | Were recommendations for policy and/or practice supported by the reported data? | Were the specific directives for new research appropriate? |
| --- | --- | --- | --- | --- | --- | --- | --- | --- | --- | --- | --- |
| Ngezi 2025 | Yes | Yes | Yes | Yes | Yes | Yes | Yes | Yes | Unclear | Yes | Yes |
| Hudson 2024 | Yes | Yes | Yes | Yes | Yes | Yes | Yes | Yes | Unclear | Yes | Yes |
| Brean 2024 | Yes | Yes | Yes | Yes | Yes | Yes | Yes | Unclear | No | Yes | Yes |
| Bassah 2024 | Yes | Yes | Yes | Yes | Yes | Yes | Yes | Yes | Unclear | Yes | Yes |
| Rodriguez-Gomez 2024 | Yes | Yes | Yes | Yes | No | Not applicable | Yes | Yes | Unclear | Yes | Yes |
| Correia-Garcia 2024 | Yes | Yes | Unclear | No | Yes | Yes | Yes | Yes | No | Yes | Yes |
| Varilek 2025 | Yes | Yes | Yes | Yes | Yes | Not applicable | Unclear | Yes | Unclear | Unclear | Yes |
| Aker 2024 | Yes | Yes | Yes | Yes | Yes | Not applicable | Unclear | Yes | Yes | Unclear | Yes |
| DeJong 2024 | Yes | Yes | Yes | Yes | Yes | Yes | Yes | Yes | Unclear | Yes | Yes |
| Sitima 2024 | Yes | Yes | Yes | Yes | Unclear | Not applicable | Yes | Yes | Not applicable | Yes | Yes |
| Coverdale 2024 | Yes | Yes | Yes | Yes | Yes | No | Yes | Unclear | Unclear | Yes | Yes |
| Ansah 2025 | Yes | Yes | Yes | Yes | Not applicable | No | Unclear | Yes | Unclear | Yes | Yes |
| Bigger 2025 | Yes | Yes | Yes | Yes | Unclear | Not applicable | Yes | Yes | Unclear | Yes | Yes |
| Kunonga 2025 | Yes | Yes | Yes | Yes | Yes | Yes | Yes | Yes | Unclear | Yes | Yes |
| Cowey 2021 | Yes | Unclear | Yes | Yes | Unclear | Unclear | Unclear | Unclear | Unclear | Yes | Yes |
| Velepucha-Iniguez 2022 | Yes | Yes | Yes | Yes | Not applicable | Not applicable | Yes | Yes | Unclear | Yes | Yes |
| Sharp 2013 | Yes | Yes | Yes | Yes | Yes | Unclear | Yes | Yes | Unclear | Yes | Yes |
| Dixon 2021 | Yes | Yes | Yes | Yes | Yes | Yes | Yes | Yes | No | Yes | Yes |
| Hart 2021 | Yes | Yes | Yes | Yes | Yes | No | Unclear | Yes | No | Yes | Yes |
| Hanan 2021 | Yes | Yes | No | No | Unclear | Unclear | Unclear | Unclear | No | Yes | Yes |
| Jones 2021 | Yes | Yes | Yes | Yes | Yes | Yes | Yes | Yes | Unclear | Yes | Yes |
| Mukherjea 2020 | No | Unclear | Yes | Yes | Yes | Not applicable | Not applicable | Unclear | No | Yes | Yes |
| LoPresti 2016 | Yes | Yes | Yes | Yes | Unclear | Unclear | Unclear | Unclear | Unclear | No | Unclear |
| Catlett 2021 | Yes | Yes | Yes | Yes | Yes | Yes | Unclear | Unclear | Unclear | Yes | Yes |
| Crist 2019 | Yes | Yes | Yes | Unclear | Unclear | Yes | Yes | Unclear | Unclear | Yes | Yes |
| Catlett 2022 | Yes | Unclear | Yes | Yes | Unclear | Unclear | Unclear | Yes | Unclear | Yes | Yes |
| Tobin 2022 | Yes | Yes | Yes | Yes | Yes | Yes | Yes | Yes | Unclear | Yes | Yes |
| Adam 2020 | Yes | Yes | Yes | Yes | Yes | No | Unclear | Yes | No | Not applicable | Yes |
| Heitner 2023 | Yes | Yes | Yes | Yes | Not applicable | Not applicable | Unclear | Yes | Unclear | Unclear | Yes |
| Hudson 2016 | Yes | Yes | Yes | Yes | Yes | Unclear | Yes | Yes | Yes | Yes | Yes |
| Connolly 2012 | Yes | Yes | Yes | Yes | Yes | Yes | Unclear | Unclear | Unclear | Unclear | Unclear |
| Morgan 2016 | Yes | Yes | Yes | Yes | Unclear | No | Unclear | Unclear | Unclear | No | Yes |
| SampedroPilegaard 2021 | No | Yes | Yes | Yes | Yes | Unclear | Yes | Yes | No |  | Yes |
| Mayeda 2019 | Yes | Yes | Yes | Yes | Yes | Yes | Unclear | Unclear | Unclear | Yes | Yes |
| Nelson 2021 | Unclear | Yes | Yes | Yes | Yes | Yes | Unclear | Yes | Yes | Yes | Yes |
| Bazargan 2021 | Yes | Yes | Unclear | Unclear | Unclear | Unclear | Unclear | Unclear | No | Yes | Yes |
| Kcomt 2017 | Yes | Yes | Yes | Yes | Unclear | Unclear | Unclear | Yes | Yes | Yes | Yes |
| Bowers 2022 | Yes | Yes | Yes | Yes | Yes | No | Unclear | Yes | Yes | Yes | Yes |
| McGowan 2022 | Yes | Yes | Yes | Yes | Yes | Yes | Yes | Yes | Unclear | Yes | Yes |
| Crooks 2023 | Yes | Yes | Yes | Yes | Yes | Yes | Yes | Yes | Yes | Yes | Yes |
| Ooko 2023 | Yes | Yes | Yes | Yes | Yes | Unclear | Not applicable | Yes | No | Yes | Not applicable |
| Wilkinson 2019 | Yes | Yes | Yes | Yes | Not applicable | Not applicable | Yes | Yes | Unclear | Yes | Yes |
| Bindley 2019 | Yes | Yes | Yes | Yes | Unclear | Not applicable | Yes | Yes | Yes | Yes | Yes |
| Wilson 2020 | Yes | Yes | Yes | Yes | Yes | Yes | Yes | Yes | Unclear | Unclear | Yes |
| Estrada 2021 | Yes | Yes | Yes | Yes | Yes | Yes | Unclear | Yes | No | Yes | Yes |
| Buchman 2023 | Yes | Yes | Yes | Unclear | Yes | Yes | Unclear | Unclear | Unclear | Yes | Yes |
| Nicholson 2023 | Yes | Yes | Yes | Yes | Yes | Unclear | Yes | Yes | No | Yes | Yes |
| Kalasapudi 2023 | Yes | Yes | Yes | Yes | Not applicable | No | No | Yes | Unclear | Yes | Yes |

**Table 3. Details of included studies**

| Study ID, Data of publication and Country in which the review conducted | Stated aim and /or objective (s) | Income of countries or primary studies | Type of review | Population group(s) | Definition of population group | Focus within dying trajectory | Number of primary studies included | Types of primary studies included | Country and continent (s) of origin of included primary studies |
| --- | --- | --- | --- | --- | --- | --- | --- | --- | --- |
| Ngezi 2025 Australia | To chart existing literature and identify gaps in the evidence base concerning PEoLC perspectives and experiences among different generations of African migrants residing outside the continent. | High | Scoping | Racialised communities | PEoLC care for African migrants residing outside the continent | Palliative; End of life | 1-10 | Mixed methods only | Mixed (more than one continent) |
| Hudson 2024  UK | To identify and review what is known about intersectionality’s impact on experiences of PEoLC, including ACP. | High | Rapid | Racialised communities | Adults or children with a terminal or life limiting illness accessing healthcare, family, friends or carers of the individuals with the terminal or life-limiting illness or healthcare professionals involved in their care | Palliative; End of life; ACP | 1-10 | Qualitative, quantitative and mixed methods | Mixed (more than one continent) |
| Brean 2024 Australia | To identify ACP tools, frameworks or guidelines available to assist vulnerable and disadvantaged adult populations; second, investigating ACP intervention  experiences, including how they were delivered; and third, assessing the effectiveness of ACP interventions based on any reported outcomes among vulnerable and disadvantaged populations, their SDM, carers  or families. The findings from the review also aim to inform practice in ACP programmes. | Middle and high | Qualitative synthesis | People experiencing homelessness  Racialised communities  People who are gender and /or sexually diverse  People in remote and rural areas; Older people, frailty and dementia  People with non-malignant disease and/or mental health disorder  People experiencing economic deprivation  People with learning disabilities  People in socio-economic deprivation  Adult vulnerable and disadvantaged populations | Not stated | ACP | 16-20 | Qualitative, quantitative and mixed methods | Mixed (more than one continent) |
| Bassah 2024 Canada | To map the literature on the palliative and end-of-life care knowledge, access gaps, and experiences of the Black Population in Canada. | High | Scoping | Racialised communities | Black Canadians living with life-limiting illnesses | Palliative; End of life | 1-10 | All study designs | North America only |
| Rodriguez-Gomez 2024 Spain | to analyse the available evidence on age- and gender-based social inequalities in access to and use of palliative care in cancer patients | Middle and high | Systematic | Age- and gender-based social inequalities | Cancer patients | Palliative | 51-60 | Qualitative research only | Mixed (more than one continent) |
| Correia-Garcia 2024 Portugal | To identify facilitators and barriers to palliative and EOLC in prisons. | High | Systematic | Incarcerated people | Not stated | Palliative; End of life | 1-10 | Qualitative research only | Mixed (more than one continent) |
| Varilek 2025 United States | To examine and describe the literature on the approaches used in the design and development of palliative and end-of-life care interventions with minoritized populations with serious illness. | High - only United States | Scoping | Racialised communities  People in remote and rural areas  People with non-malignant disease and/or mental health disorder | Minoritized populations with serious illness.  -Minoritized populations including non-white, non-English speaking, gender minority, incarcerated, and ill-housed/unhoused, and being from rural and frontier locations | Palliative; End of life | 30-40 | All study designs | North America only |
| Aker 2024 UK | To identify how older people from minority ethnic groups access and utilise palliative and end of life care, and how does this vary between different ethnicities, countries, and health conditions. | High | Scoping | Racialised communities  Older people, frailty and dementia | Older people from minority ethnic groups in any country, who were receiving (or had received) generalist or specialist palliative or end of life care for any health condition; informal carers; and healthcare professionals. | Palliative; End of life | 21-25 | Qualitative research only including qualitative data of mixed methods research | Mixed (more than one continent) |
| DeJong 2024 Canada | To identify the palliative care use among 2SLGBTQIA+ people and identify common themes in the literature. | High | Scoping | People who are gender and /or sexually diverse  2SLGBTQIA+ | The acronym 2SLGBTQIA+ includes sexual and gender minorities who are Two-Spirit, Lesbian, Gay, Bisexual, Transgender/Trans, Queer/Questioning, Inter-sexual, Asexual/Aromantic/Agender and all others | Palliative; End of life | 31-35 | Qualitative, quantitative and mixed methods | Mixed (more than one continent) |
| Sitima 2024 Portugal | To systematically examine (1) the primary contributors to inequity in accessing PC; (2) the key barriers to achieving equitable access to PC; (3) the essential recommendations for addressing these issues | High | Scoping | Racialised communities  People who are gender and /or sexually diverse  People in remote and rural areas  People experiencing economic deprivation  People in socio-economic deprivation | Ethnic/racial discrimination, geographical, and socioeconomic discrepancies as the primary contributors to inequity in accessing PEoLC services | Palliative; End of life | 11-15 | All study designs | Mixed (more than one continent) |
| Coverdale 2024  UK | To identify interventions and models of care used to address the palliative and end of life care needs of homeless adults, and to determine their  effectiveness. | High | Systematic | Homeless | It involves deprivation across a number of different dimensions—physiological (lack of bodily comfort or warmth), emotional (lack of love or joy), territorial (lack of privacy), ontological (lack of rootedness in the world, and anomie) and spiritual (lack of hope, lack of purpose) - Somerville Definition | Palliative; End of life | 16-20 | All study designs | Mixed (more than one continent) |
| Ansah 2025 United States | To identify and describe what is known about PC use among Black HM patients in the United States. | High | Scoping | Racialised communities | Black/African American with Haematological Malignancy (18 years and above) | Palliative; End of life | 1-10 | Quantitative research only | North America only |
| Bigger 2025 United States | To identify the state of the literature on language interpreting in palliative care and to examine inclusion of stakeholders in dissemination products. | High | Scoping | Language justice | Not stated | Palliative | 31-35 | All study designs | North America only |
| Kunonga 2025  UK | This rapid systematic review aims to explore ethnic inequalities in palliative care prescribing amongst adults  residing in high-income countries. | High | Integrative | Racialised communities | Adults aged 18 +with a life-limiting illness with a focus on ethnic minorities receiving palliative care from generalists or specialists’ providers and taking medicines for symptom control | Palliative | 1-10 | Quantitative research only | North America only |
| Cowey 2021  UK | Our aim was to narratively review research on palliative care and stroke, published since 2015. | High | Narratively review | Palliative care and stroke | Not stated | Palliative; End of life | 31-35 | Quantitative research only | Mixed (more than one continent) |
| Velepucha-Iniguez 2022 Ecuador (first two authors) and US (last author) | The main aim of this scoping review was to identify possible barriers to PC delivery to patients with Intellectual disabilities. | High | Scoping | People with learning disabilities  Patients With Intellectual Disability | Patients With Intellectual Disability Studies with participants with intellectual disabilities or caregivers and healthcare workers related to patients with intellectual disabilities were included. | Palliative | 21-25 | All study designs | Mixed (more than one continent) |
| Sharp 2013  UK | To investigate the attitudes of the public and healthcare professionals to advance care planning discussions with frail and older people. | High | Systematic | Older people, frailty and dementia | The public and healthcare professionals to discussions about end-of-life care with frail and older individuals with no overriding diagnosis. | End of life | 26-30 | All study designs | Mixed (more than one continent) |
| Dixon 2021  UK | To address the palliative and end of life care experiences, views and needs within travelling communities. | High | Qualitative synthesis | People in socio-economic deprivation  Gypsy, Traveller and Roma | Gypsy, Traveller and Roma communities | Palliative; End of life | 1-10 | Qualitative research only | Europe |
| Hart 2021 United States | To examine the literature related to EOL interventions culturally targeted for African Americans. | High | Scoping | Racialised communities | African Americans | End of life | 11-15 | Quantitative research only | North America only |
| Hanan 2021 United States | To identify factors associated with the use of hospice services by individuals with Severe Persistent Mental Illness. | High | Integrative | People with non-malignant disease and/or mental health disorder  Severe Persistent Mental Illness | SPMI describes a subset of mental illnesses that typically have their onset in early adulthood and have profound effects on relationships, capacity for educational attainment and employability, and social functioning. | Hospice use | 1-10 | All study designs | Mixed (more than one continent) |
| Jones 2021 United States | To summarize and evaluate existing palliative and end-of-life care interventions that aim to improve outcomes for racial and ethnic underrepresented populations in the United States. | High | Systematic | Racialised communities | Studies on racial/ethnic underrepresented populations specifically | Palliative; End of life; ACP | 16-20 | Quantitative research only | North America only |
| Mukherjea 2020  United States | To understand of whether and how palliative care and other EoL interventions are accessible to and utilized by diverse Asian American sub- groups. | High | Scoping | Racialised communities | Asian Americans including Cambodia, China, India, Japan, Korea, Malaysia, Pakistan, the Philip-pine Islands, Thailand, and Vietnam.” | Palliative; End of life | 21-25 | Qualitative, quantitative and mixed methods | North America only |
| LoPresti 2016  United States | To examine the relationship between ethnic minority status and the use of EOL care among people with cancer. | High | Systematic | Racialised communities | Ethnic minority group | End of life | 21-25 | All study designs | North America only |
| Catlett 2021 United States | To examine the literature highlighting participant responses to ACP and EOL care literacy initiatives in AA faith communities. | High | Systematic | Racialised communities | African American parishioners | End of life; ACP | 1-10 | Quantitative and Qualitative research | North America only |
| Crist 2019 United States | To better understand this disparity in Mexican Americans caregiving families’ end-of-life care decisions. | Low; Middle and high | Integrative | Racialised communities | Hispanics, Latinos, or Mexican Americans.  At least 50 years old and/or families. | End of life | 21-25 | All study designs | South America only |
| Catlett 2022 United States | To synthesize recent studies addressing the healthcare needs and assets of gender diverse older adults living in  the United States (US), and (2) to summarize themes and subthemes relevant to improving healthcare practice, policy, and research for  this population. | High | Integrative | People who are gender and /or sexually diverse | Aging members of the Lesbian, Gay, Bisexual, Transgender, Queer/Questioning, Intersex, and Asexual/Agender (LGBTQIA+) | End of life | 31-35 | All study designs | North America only |
| Tobin 2022  UK | To systematically review and synthesise the peer-reviewed literature concerning referral to or receipt of hospice care with regards to eight characteristics: diagnosis and comorbid conditions; age; gender and sexuality; marital status; ethnicity and religion; geographical factors including rurality and distance to hospice; socioeconomic factors; other characteristics | High | Qualitative synthesis | Racialised communities | Adult population  We restricted studies to those from countries with similar models of hospice care and health service provision: UK, Canada, Australia and New Zealand. | Palliative | 130 | All study designs | Mixed (more than one continent) |
| Adam 2020  UK | To identify the palliative care needs of adults with intellectual dis-abilities and the barriers and facilitators this population face in accessing palliative care. | High | Systematic | People with learning disabilities | Learning disability is present when the following three criteria are met: a significantly reduced ability to understand new or complex information and to learn and apply new skills (impaired intelligence); a reduced ability to cope independently (impaired social functioning); and beginning before adulthood, with a lasting effect on development. | Palliative | 51-60 | All study designs | Mixed (more than one continent) |
| Heitner  2023  United States | To identify and characterize the literature on the experiences of Black patients with serious illness across multiple domains − physical, spiritual, emotional, cultural, and healthcare utilization. | High | Scoping | Racialised communities | Black Patients with Serious Illness in the United States. | Palliative; End of life; Bereavement | 160 | All study designs | North America only |
| Hudson 2016  UK | To explore the perspective of homeless people and those working to support them, current challenges to palliative care access and provision, in addition to suggestions for what may improve palliative care for this population. | High | Qualitative synthesis | People experiencing homelessness | Homeless individuals as living on the street, using temporary accommodation or hostels. | Palliative; End of life | 11-15 | Qualitative research only | Mixed (more than one continent) |
| Connolly 2012  UK | To examine (1) the differences in interventions used at the end of life, including life-sustaining medical interventions, palliative care, and the preferences for these and (2) the potential barriers to providing good end-of-life care to people with dementia from ethnic minorities, including attitudes and experiences of people with dementia and their formal and informal caregivers. | High - only United States | Systematic | Racialised communities  Older people, frailty and dementia | People with dementia or severe cognitive impairment or their caregivers with ethnic minority people as a main or a specific subgroup and an outcome involving end-of-life care | Palliative; End of life; ACP | 16-20 | Quantitative and qualitative research | North America only |
| Morgan 2016  New Zealand | To identify and synthesise the empirical literature  from the past 20 years that examines the relation- ship between gender and family caregiving at end  of life of patients over 65 years in order to explore  how gender as a social construction impacts on caregivers’ experiences | High | Systematic | People who are gender and /or sexually diverse | Gender is to be understood as ‘the social, cultural and symbolic construction of femininity and masculinity in any given society’ | End of life | 19 | All study designs | Mixed (more than one continent) |
| SampedroPilegaard 2021 Denmark | To map existing research of rehabilitation and palliative care for patients with advanced cancer who are socioeconomically disadvantaged | High | Scoping | People in socio-economic deprivation | Socioeconomic disadvantage is often defined in the literature by socioeconomic position, where income, educational level and occupational status are the most commonly used indicators | Palliative (rehabilitation) | 11-15 | All study designs | Mixed (more than one continent) |
| Mayeda 2019 United States | To evaluate barriers to palliative care in ethnic minorities. | High | Systematic | Racialised communities | Racial/ethnic or underserved minorities in the healthcare field | Palliative; End of life | 1-10 | Quantitative research only | North America only |
| Nelson 2021 United States | To synthesize current literature on disparities in access to hospice and palliative care, highlight the range of sociodemographic groups affected by these inequities, characterize the domains of access addressed, and outline implications for research, policy, and clinical practice. | High | Integrative | Racialised communities  People in socio-economic deprivation | Socially and economically disadvantaged groups of people, such as Blacks, Hispanics, and Native Americans. Individuals “situated on lower rungs of social hierarchies of power,” such as individuals with low-income or without a permanent residence. | Palliative | 16-20 | Qualitative, quantitative and mixed methods | North America only |
| Bazargan 2021  United States | To summarize the current published research and literature on disparities in palliative and hospice care and completion of advance care planning and directives among non-Hispanic Black. | High | Scoping | Racialised communities | Blacks/African American or non-Hispanic Blacks | Palliative; End of life; Bereavement | 147 | All study designs | North America only |
| Kcomt 2017 Canada | To examine the prevalence of EOL preparatory behaviours among LGBT people, with particular focus on transgender individuals. | High | Integrative | People who are gender and /or sexually diverse | Lesbian, gay, bisexual, and transgender (LGBT) people | Palliative; End of life | 26-30 | Quantitative research only | Mixed (more than one continent) |
| Bowers 2022  UK | To summarise the current global evidence from developed countries on end-of-life experience for those living with socio-economic deprivation. | High | Integrative | People experiencing economic deprivation | People with socio-economic deprivation in developed world (high income countries) | End of life | 40 | Qualitative, quantitative and mixed methods | Mixed (more than one continent) |
| McGowan 2022  United States | To systematically analyse the literature to assess the prevalence of racial disparities in the ICU. | High - only United States | Systematic | Racialised communities | Patients in medical, surgical, cardiac, and neurology ICUs were included | Palliative (ICU) | 21-25 | Quantitative research only | North America only |
| Crooks  2023  UK | To examine differences in the presence of documented ACP in individuals’ care records for people with advanced disease by ethnic group and identify patient and clinician related factors contributing to this. | High; Middle and low | Mixed-methods systematic | Racialised communities | • Adults with any advanced, incurable, life-span limiting  condition or disease; multi-morbidity (so long as at least one condition is advanced); ‘serious illnesses’ that have led to palliative care consultation • Proxy discussions of any ‘real patients’ (e.g., by a relative/ carer or healthcare professional) where the patient otherwise  meets the inclusion criteria • (For RQ2) Any healthcare professional (e.g., consultant, doc- tor, nursing staff ) • Any self-defined ethnicity | ACP | 31-35 | Quantitative and qualitative research | Mixed (more than one continent) |
| Ooko 2023 South Africa | To understand the geographic accessibility, availability, financial accessibility, and acceptability of palliative care by patients with advanced cervical cancer and their families | Middle and low | Systematic | Patients with cervical cancer and their families. | Women diagnosed with advanced cancer of the uterine cervix (advanced cancer | Palliative; End of life | 11-15 | All study designs | Mixed (more than one continent) |
| Wilkinson 2019  UK | (1) To identify the evidence base of inequalities in the outcomes of end-stage kidney disease for ethnic minorities; (2) to map the international literature; and (3) to identify gaps and make recommendations for research and policy towards reducing and preventing inequalities in kidney care in the UK and globally | High; Low | Scoping | Racialised communities  People with non-malignant disease and/or mental health disorder  End-stage kidney disease (ESKD) for ethnic minorities | End-stage kidney disease (ESKD) for ethnic minorities | End of life | 112 | All study designs | Mixed (more than one continent) |
| Bindley 2019 Australia | To reflexively identify themes pertaining to both social and structural inequities as features of structural vulnerability in published research; considering the consequences for individuals bereaved following life-limiting illness, and situated within systems and networks underpinned by norms and values with the potential to shape experiences of structural vulnerability and disadvantage. | High | Scoping | Racialised communities  People who are gender and /or sexually diverse  Older people, frailty and dementia  People experiencing economic deprivation | 'bereaved populations' | Bereavement | 11-15 | All study designs | Mixed (more than one continent) |
| Wilson 2020 (2019 online)  UK | The aim of this study was to systematically review the empirical evidence assessing end-of-life care and place of death for people with serious mental illness.  It was intended that exploring the health care people with serious mental illness receive at the end of life would highlight any end-of-life care inequalities this population face. Specifically, we aimed to explore what healthcare services people with serious mental illness utilised in the last 12 months of life and where deaths occurred | High | Systematic | People with non-malignant disease and/or mental health disorder  People with serious mental illness | Adults (18+years) with a diagnosis of serious mental illness, who had died or were in the last year of life | End of life | 21-25 | Quantitative research only | Mixed (more than one continent) |
| Estrada 2021  United States | We aim to identify differences in Nursing Home EOL care for racial/ethnic minority residents. The aim of this systematic review was to examine the differences in EOL care for racial/ethnic minority Nursing Home residents. | High | Systematic | Older people, frailty and dementia | Nursing Home residents over 60 years old, terminally ill or approaching end of life. | End of life | 16-20 | Quantitative research only | North America only |
| Buchman 2023  Canada | To explore what is known about communicable disease epidemics and pandemics, palliative care, and people who use drugs. | High | Scoping | People experiencing homelessness  People experiencing economic deprivation | Adults (⩾18 years of age) who use drugs (including those who receive treatment for substance use disorders and use prescription drugs and/or unregulated substances) and have life-limiting illnesses (e.g. advanced cancer). | Palliative | 41-45 | Quantitative and qualitative research | Mixed (more than one continent) |
| Nicholson 2023  UK | To understand the palliative care needs of community-dwelling people aged ⩾60 living with multimorbidity in the last 2 years  of life | High | Scoping | Older people, frailty and dementia | Older (over 60 years of age) with multimorbidity and frailty | Palliative; End of life | 26-30 | All study designs | Mixed (more than one continent) |
| Kalasapudi  2023  United States | To identify racial, ethnic, and sex disparities pertaining to the diagnosis and management of haemorrhagic stroke. | High -only United States | Scoping | Racialised communities  People who are gender and /or sexually diverse  People with non-malignant disease and/or mental health disorder | adults with spontaneous intracerebral haemorrhage (ICH) or aneurysmal subarachnoid haemorrhage (SAH) in the US. | Palliative; End of life | 51-60 | Quantitative research only | North America only |

PEoLC: Palliative and end of life care, ACP: Advance care planning
